# Immune Response in Mice Immunized with Chimeric H1 Antigens

**DOI:** 10.3390/vaccines9101182

**Published:** 2021-10-15

**Authors:** Erasmus Nikoi Kotey, William Kwabena Ampofo, Rebecca Daines, Jean-Remy Sadeyen, Munir Iqbal, Osbourne Quaye

**Affiliations:** 1 West African Centre for Cell Biology of Infectious Pathogens (WACCBIP), University of Ghana, Legon, Accra 23321, Ghana; enkotey003@st.ug.edu.gh; 2 Department of Biochemistry, Cell & Molecular Biology, University of Ghana, Legon, Accra 23321, Ghana; 3 Noguchi Memorial Institute for Medical Research, University of Ghana, Legon, Accra 23321, Ghana; wampofo@noguchi.ug.edu.gh; 4 The Pirbright Institute, Ash Road, Pirbright, Woking GU24 0NF, UK; rebecca.daines@pirbright.ac.uk (R.D.); jean-remy.sadeyen@pirbright.ac.uk (J.-R.S.); munir.iqbal@pirbright.ac.uk (M.I.); 5 Department of Pathobiology and Population Sciences, Royal Veterinary College, Hawkshead Lane, North Mymms, Hatfield AL9 7TA, UK

**Keywords:** universal influenza vaccine, broad-reactive antibodies, chimeric haemagglutinin, cross-reactivity, seasonal influenza, therapeutic, prophylactic

## Abstract

Identification of a universal influenza vaccine candidate has remained a global challenge for both humans and animals. This study describes an approach that uses consensus sequence building to generate chimeric HAs (cHAs): two resultant H1 HA-based chimeras comprising of conserved sequences (within several areas spanning the head and stalk regions) of H1 and H5 or H9 HAs. These cHAs expressed in *Drosophila* cells (S2) were used to immunize mice. All immunized mice were protected from an infectious H1 virus challenge. Seroconverted mice sera to the H1 cHAs inhibited both the challenge virus and an H5 virus isolate by haemagglutination inhibition (HI) assay. These findings further emphasize that cHAs induce cross-reactive antibodies against conserved areas of both head and stalk regions of the seasonal influenza A (H1N1) pdm09 virus’ HA and holds potential for further development of a universal influenza vaccine.

## 1. Introduction

Influenza is a contagious viral disease associated with epidemics estimated to cause about half-a-million mortalities and millions of morbidities, yearly [1]. In retrospect, the last influenza pandemic, for instance, led to between a hundred and five hundred thousand cases of mortalities alone [2], thereby posing immense concern about what the next influenza pandemic will cause.

Twice yearly, the WHO organizes expert meetings to deliberate on which viruses have been advanced for vaccine consideration for both the northern and southern hemispheres. These viruses are mainly those captured during annual surveillance and are used to predict the antigenicity of viruses in the forthcoming influenza season. Selected candidate vaccine viruses thus antigenically correspond to viruses detected in circulation for a specific hemisphere. Seasonal vaccines are efficacious when selected candidate vaccines match circulating influenza viruses [3,4]. Seasonal influenza vaccines induce protective humoral responses targeting the immunodominant “head” region of the influenza virus haemagglutinin (HA) protein. However, the HA is subject to continuous evolution, which often renders the vaccine and circulating virus antigenically mismatched, resulting in suboptimal vaccine effectiveness [5,6].

The stalk regions of the HAs are relatively more sequence stable than the head region to evolutionary pressure, albeit functionally less immunodominant since most vaccination regimens hardly induce stalk-reactive antibodies [7]. However, stalk-specific antibodies can cross-react with diverse strains and subtypes of influenza viruses mediating antiviral functions such as antibody-dependent cellular cytotoxicity (ADCC), antibody-dependent cellular phagocytosis (ADCP), or and complement-dependent cytotoxicity (CDC) [8,9]. Thus, HA antigens inducing potent cross-reactive antibodies to the stalk or whole HA may offer a potential universal vaccine candidate.

One approach to induce stalk-directed immunity is via the sequential administration of chimeric haemagglutinin-based (cHA) vaccines, comprising seasonal influenza virus HA stalk and the heads of avian subtypes to which human immunity is unfamiliar. This approach has recently displayed an immune refocusing ability of antibody responses to the stalk domain of the HA [10,11,12,13]. Therefore, enhancing the cHA-based vaccine design to induce broadly reactive antibodies targeting all available conserved regions or epitopes on the HA will prove more useful for the development of a universal influenza vaccine if it meets desired efficacy limits and is associated with a relatively longer term of protection. A candidate of the sort will even give room for fine tuning of epitopes with the potential to induce antibodies carrying antiviral activity against most, if not all, susceptible seasonal influenza virus strains.

This study aimed at exploring the ability of H1-based cHAs designed by consensus building, to induce cross-reactive antibodies. In accordance with previously described cHAs, H5 and H9 HAs, being members of the group 1 HA (to which H1 HA also belongs), were preferentially selected as the source of foreign sequences for the generation of the H1-based cHAs. Therefore, two cHAs (H1/H5 HA and H1/H9 HA) predominantly made up of conserved regions of H1 HAs were designed in silico. Expressed proteins of these cHAs were used to immunize mice. Mice weights were generally stable upon challenge with an H1 virus isolate, confirming induction of protective anti-cHAs antibodies. Seroconverted sera from the immunized mice demonstrated cross-reactivity to an H5 virus isolate, positing cHAs-based vaccines as potentially useful means for the fine-tuning of HA antigens for use in the development of a universal influenza vaccine candidate.

## 2. Materials and Methods

### 2.1. HA Sequence Selection and Consensus Sequence cHA Construction In Silico

Several numbers of HA nucleotide sequences, i.e., 1156 H1 HAs (comprising both former H1 and H1pdm09), 76 H5 HAs, and 3771 H9 HAs (including H5 and H9 strains isolated from human hosts), were preferentially selected from the Influenza Research Database [14] and the GISAID. Downloaded sequences were viewed using the BioEdit Sequence Alignment Editor software [15]. For each set of HA sequences downloaded, multiple alignment analyses were performed by uploading files onto the online MAFFT software [16] and run at default parameters. Each multiple aligned sequence file was exported to the BioEdit software for editing (i.e., trimming of untranslated regions of sequences and retaining the start and stop in order of 5′ to 3′). Subsequently, each of the nucleotide sequences that comprise the multiple aligned sequences was translated into amino acid sequences and followed by the generation of a consensus sequence, one sequence representing all multiple aligned sequences. Multiple aligned protein sequences were uploaded again unto the MAFFT server, where the phylogenetic analysis was performed: phylogenetic trees were viewed on the java-enabled archaeopteryx software or MEGA 7 software [17].

Due to the diversity of sequences for each type of consensus HA, the resulting amino acids had gaps that represented polymorphic sites. Considering that chimeric HAs have shown tremendous results in terms of antibody refocusing to the less immunodominant and conserved HA stalk domain [18,19], chimeric HAs were developed by aligning H1 HA consensus amino acid sequences with H5 or H9 consensuses to form chimeric HAs designated as H1/H5 HA (cHA-C) and H1/H9 (cHA-E), respectively. BLASTp analysis on the NCBI server was performed to confirm that cHAs were predominantly seasonal influenza H1-like [20].

### 2.2. cHA Structural and Functional Predictions

Based on encouraging BLASTp results, structure, and/or functional predictions were made using the I-TASSER platform [21]. The closest structural models predicted were obtained from the Protein Database (https://www.rcsb.org/, accessed on 1 January 2019) and visualized in Pymol version 4.6.0 [22]. Some key amino acids that make up the receptor binding constellation (including residues at 129, 158, 163, and 165) of each of the cHAs had been altered due to the consensus building approach used to design the cHAs. For cHA-C, positions 129, 163 and 165 remained unaltered. Only the amino acid at position 158 was substituted with leucine (L) (i.e., G/E/D/N 158 L). cHA-E on the other hand, had amino acids at 158 and 163 drastically altered to L and P, (i.e., G/E/D/N 158 L; and K/N 163 P). At position 165, there was a replacement with an amino acid (i.e., Q) that has similar chemical characteristics as N (i.e., S/K/N 165 Q). These subtle substitutions, therefore, indicate a potentially altered degree of receptor binding of the cHAs.

### 2.3. cHA B-Cell Epitope and Antigenic Predictions

Immune parameter predictions, such as B-cell epitope prediction (performed on the webserver http://www.cbs.dtu.dk/services/BepiPred-2.0/, accessed on 5 January 2019) and antigenicity prediction (performed on the Immune Epitope Database and analysis resource platform, based on Kolaskar and Tongaonkar algorithms) were made [23]. Briefly, “FASTA” formatted amino acid sequences were deposited on the afore-specified web interface and epitope probability scores were recorded at the default threshold of 0.50 for the detection of B-cell epitopes. Means of the scores for control HA (the 2018/2019 candidate vaccine viruses’ HAs: A/Michigan/45/2015 (H1N1) pdm09-like virus, CVV-M) and cHAs were further assessed for significant differences by one-way ANOVA. Predicted antigenic peptides were analyzed: common peptides were eliminated, whilst unique peptides present in each of the HA sequences were documented.

### 2.4. cHA Design

The cHAs constructs were designed by replacing the natural signal peptide of HA (amino acids 1–15), and transmembrane domains (TM) amino acids 536 to 572 were identified with the aid of the SignalP-5.0 server [24] and the ExPASy TMpred tool (Swiss Institute of Bioinformatics, Lausanne, Swiss), respectively. The signal peptide was replaced with the *Drosophila* binding immunoglobulin protein (BiP) signal peptide, and the TM was replaced with the “foldon” sequence (from bacteriophage T4 fibritin for trimerization of HA) [25]. This was performed to produce soluble secreted trimeric HA antigens in the *Drosophila* S2 cell culture medium [26,27,28,29]. The c-terminus of HA sequence also contained additional 12 amino acids encoding V5 tag and four amino acids encoding C-tags, both aiding detection and purification of expressed proteins.

The HA amino acid sequences were backtranslated into nucleotide sequences using the online EMBOSS Backtranseq tools (European Bioinformatics Institute, Cambridge, UK). By translating again with the ExPASy translate tool (Swiss Institute of Bioinformatics, Swiss), the HA expression cassette coding sequences were codon-optimized for expression in *Drosophila* cells. These cassettes were cloned in silico into modified pExpreS2-1 vectors between the signal peptide (BIP) and foldon-V5-tag sequences, using SmaI-NotI restriction sites in the 5′-3′ direction, respectively, to determine the integrity of the HA open reading frame.

### 2.5. cHAs Cloning and Transfection

Correct in-frame HA nucleotide sequences were synthesized by GeneArt (Thermo Fisher, Leicestershire, UK). Synthesized HAs (borne on GeneArt plasmids) were reconstituted with nuclease-free water for subsequent cloning and subcloning onto the pExpreS2V1 plasmid (ExpreS2ion Biotechnologies, Hørsholm, Denmark). Successfully cloned plasmids were purified and used to transfect S2-cells. Briefly, 5 mL Schneider’s S2 medium (supplement with 10% fetal calf serum, FCS) was seeded with about 1.0 × 10^6^ cells and incubated for up to 3 days at 28 °C, in the absence of CO_2_, until a microscope-aided observation of confluence of about 80%. The calcium phosphate transfection method was applied to transfect the confluent cells; a green fluorescent protein (GFP) control was set up to check for the efficiency of the transfection process. Transfected cells were incubated at 28 °C. FCS-supplemented S2 medium was changed over transfected cells 24 h post-transfection and was further incubated for 72 h, until antibiotic selection, which involved the use of 750 µg/mL zeocin in FCS-supplemented S2 medium. Concurrently, supernatant over the cells was harvested and used to assay for protein expression by either ELISA or Western blot using an anti-V5 primary mouse monoclonal antibody (ThermoFisher Scientific, Waltham, MA, USA) and an anti-mouse polyclonal secondary antibody conjugated to either HRP or green infrared dye (800 CW) (LI-COR Biosciences, GmbH, Lincoln, NE, USA), respectively.

#### 2.5.1. Purification of cHAs and Vaccine Preparation

Confirmed cHA expressing-S2 cells’ supernatants were stored after every three to four days, during medium change. Cells were subsequently grown from T25 cm^2^ flasks through T150 cm^2^ flasks to roller bottles, in which proteins were massively produced with FCS-free Excel 420 medium (Merck, Darmstadt, Germany). Supernatants were pooled for each protein expression setup and stored at 4 °C before purification.

Briefly, culture supernatants containing recombinant HA proteins with c-terminal EPEA sequence (c-tag) were filtered using 0.22 µm filters (by Millipore, Burlington, MA, USA), dialyzed in PBS (with no Ca + or Mg +) (PBS-) overnight at 4 °C, and purified on a CaptureSelect™ C-tag Affinity Matrix (ThermoFisher Scientific) column as per supplier instructions. Bound protein was eluted with 20 mM PBS, containing 2M magnesium chloride, pH 7.2. Eluted fractions containing proteins were further dialyzed in PBS-. Finally, proteins were concentrated (using the Amicon ultrafilters-3k, Millipore), quantitated using the BCA assay, adjusted to 30 µg/mL, and stored at −85 °C.

#### 2.5.2. HA Antigens’ Haemagglutination (HA) Activity Assessment

Using a 96-well plate, 50 µL of the concentrated cHAs (~30 µg/mL) were serially diluted two-fold and mixed with an equal volume of 1% turkey red blood cells. Plates were gently agitated, incubated for 30 min at room temperature, and observed for haemagglutination. Experiments were performed in triplicate, and each was controlled using a lab-isolated influenza virus.

### 2.6. Haemagglutination Inhibition (HI) Assay

Levels of HA antigen-specific antibodies in sera from naïve, vaccinated and challenged mice were measured using standard HI assays [30]. The HI assays were performed using 4-*HA* units (HAU) of H1N1 pdm09 virus reference antigens sourced from WHO Influenza reference laboratories (WHO serology kit, IRR). The serum collected from each mouse was treated with four parts of receptor destroying enzyme (RDE) (WHO serology kit, IRR), incubated on a heated block at 37 °C overnight. The enzyme reaction was stopped by the addition of five parts of physiological saline and incubated at 56 °C for 30 min. This makes up a serum dilution of 1:10, which was further serially diluted (in two-fold) with PBS in a 96-well plate. The 96-well plate containing 25 μL of diluted antisera in each well was mixed with 25 μL of 4HAU-standardized H1N1 pdm09 virus antigen and 50 μL of 0.75% turkey red blood cells. The plates were tapped gently to mix, and the HI titres were recorded after 45 min room temperature incubation.

### 2.7. Immunization

**Figure 1:**
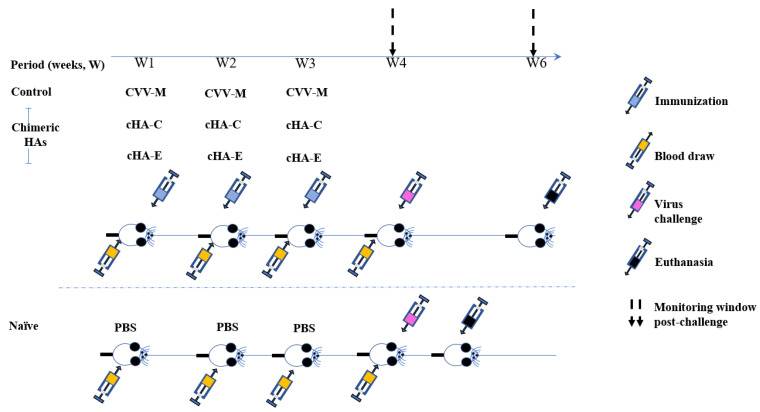
A schematic mice immunization and challenge regimen.

Six- to eight-week-old International Cancer Research (ICR) mice (*n* = 20) showing no detectable levels of immune exposure to an influenza A (H1N1) pdm09 influenza virus (by HI assay) were divided into four groups of five mice. Three groups of the mice were intraperitoneally immunized with 30 µg (in PBS) of either one of the non-adjuvanted cHAs antigens (cHA-C and cHA-E) or the CVV- M antigen (positive control). The naïve unvaccinated control group (U) received PBS only. Boost vaccination was performed with the same amount of the priming antigen after a week for three consecutive times to enhance antigen delivery and corresponding immune response. Before the boosting periods, blood was sampled via tail clipping of each mouse, pooled (to increase the amount serum), and assessed for seroconversion amongst the test groups and controls. At the same time, blood was drawn from the naïve negative control mice for continuous monitoring of un-intended virus exposure (illustrated in Figure 1). Sera were drawn from mice on the 3rd week (3D sera) before the virus challenge on the 4th week. Collected blood specimens from each group were pooled together for assessment of the presence of neutralizing antibodies by the HI assays.

This figure is the summary of the immunization and virus challenge work up. Four groups, each comprising five International Cancer Research (ICR) immunocompetent mice: All four groups (i.e., cHA-C, cHA-E, CVV-M, and U) were intraperitoneally immunized with 30 µg/mL (formulated in PBS) each of cH1/5 HA, cH1/9 and Michigan Candidate vaccine virus HA, and the U was the naïve controls for the group 1 HAs, respectively. For weeks two and three, each mouse (excluding the naïve groups) was boosted with 30 µg/mL per week, of the respective construct. At the start (i.e., from week one (W1), blood was drawn from each mouse and was pooled at the group level for exposure-to-influenza virus checking. From W2 to W4, group-specific pooled blood specimens were compared with the baseline blood to estimate seroconversion by haemagglutinin inhibition assay. Similarly, each member of groups C, E, M, and U was challenged with 1.0 × 10^7^ PFU of A/England/195/2009 influenza virus (donated by Prof Wendy Barclay from the Imperial College of London). The permit to work on the mice was obtained from the University of Ghana Institutional Animal Care and Use Committee (UG-IACUC) under the license (UG-IACUC 006/19–20) and the experiment was performed at the Centre for Plant Medicine Research (CPMR).

#### Virus Challenge

Mice were challenged with 50 µL of 1.0 × 10^7^ plaque forming units (PFU) of A/England/195/2009 (H1N1) pdm09 intraperitoneally at four-week post-vaccination, adapting a previously described procedure that favors either induction of immune activity of peritoneal cells or enhances antibody interaction with viruses in the case of a pre-immunized state [31]. Mice weights were monitored daily for 14 days after the challenge. Mice that attained a weight loss >30% of their initial were euthanized during monitoring. Mean differences in weight loss were assessed for significant differences by ANOVA using the GraphPad Prism 8.0.1.

Furthermore, by day three post-challenge, one mouse per group was euthanized and lungs were harvested and subjected to real-time RT-PCR testing to determine the viral RNA titres in lungs homogenates adopting the CDC protocol influenza H1 subtyping protocol [32]. Briefly, homogenates of lungs were centrifuged at 15,000 rpm for 15 min. About 140 µL of supernatant was collected and RNA isolated with the QIAamp^®^ Viral RNA Minikit (Qiagen, Hilden, Germany) according to the manufacturer’s protocol. Using the Applied Biosystems ^TM^ AgPath-ID ™ One-Step RT-PCR reagents, a master-mix was prepared using influenza A-specific primers and probes (IRR) according to the CDC protocol. About 5 µL of RNA extracts were added to the 20 µL of the master-mix and tested in duplicate on the ABI 7500 real-time PCR device. The threshold cycle (Ct) values were log-transformed and reciprocated to provide a sense of viral load in lungs of each mouse per group.

## 3. Results

### 3.1. Conceptual Design of the H1-Based cHAs

**Figure 2:**
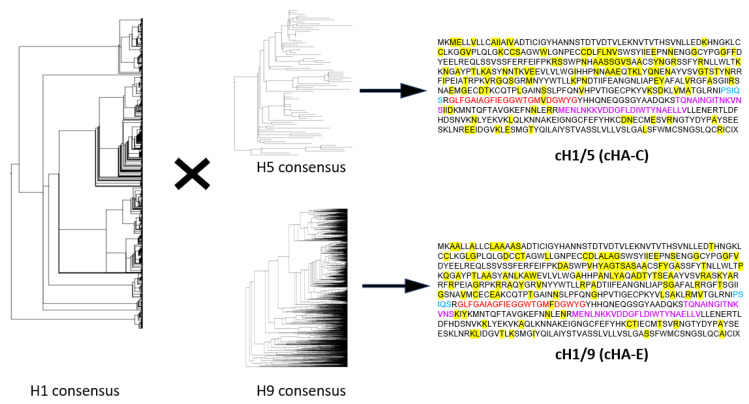
Chimeric HA generation through consensus building of H1 and H5 or H9 HAs.

Phylogenetic analyses for diverse H1, H5, and H9 HA sequences confirm the diversity of sequences selected from the Influenza Research Database (IRD) and Global Initiative on Sharing All Influenza Data (GISAID) online repository (i.e., phylograms in Figure 2). Diverse sequences of each subtype of HA were all aligned using the MAFFT software, followed by a consensus-building (by BioEdit). Primarily, the H1 consensus generated was used as the parental sequence (Figure 2), from which two chimeras (H1/H5 HA (cHA-C) and H1/H9 HA (cHA-E)) were generated by filling in polymorphic regions with conserved amino sequences sourced from the H5 or H9 consensuses, respectively (Figure 2).

Consensus sequences were generated from H1 (over 1000), H5 (76), and H9 (over 3000) sequences. Sequences from the H5 or H9 consensuses were used to fill in spaces within the H1 consensus to generate two chimeric HA molecules: cHA-C (cH1/5) and cHA-E (cH1/9), respectively. These chimeras reveal both the conserved sequences (represented as amino acids in black fonts) and polymorphic sites (represented by the yellow highlighted sequences serving as areas into which H5 or H9 sourced amino acids were introduced) of the H1 HA. The cleavage site (blue fonts), fusion peptide (red fonts) and heptad repeats (purple fonts) are key conserved regions.

### 3.2. cHAs Are Relatively Similar in Structure and Function to a Typical HA

**Figure 3:**
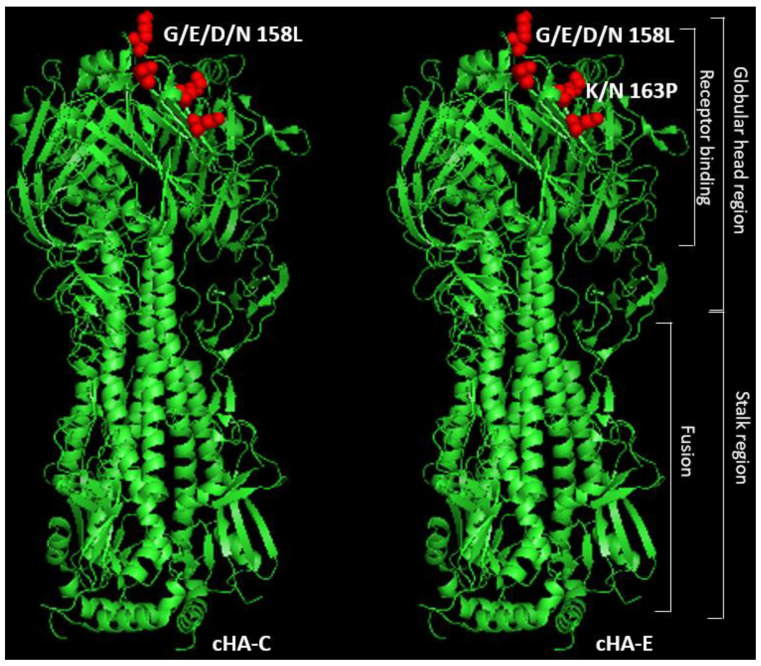
Predicted structures of the in silico synthesized cHAs.

Structural predictions of each of the cHAs by the I-TASSER online platform indicated about 80% structural similarity as well as a 70% potential to be similar in function as an influenza HA. These predictions were based on TM-scores, which predict structural similarity to a hit protein template, and C-scores, a parameter that predicts that the likelihood of function of the protein being assessed (Table 1 and Figure 3). Model structures predicted were similar to an HA type 16 (a type which categorizes with group 1 HAs, where H1 HA is a member) crystal structure (42f3) in the Protein Database [33]. Sequence analyses and visualization of the structure was by aid of Pymol [34], the platform that also afforded the visual representation of key amino acid substitutions within the specific regions of the receptor binding domains of the cHAs: “G/E/D/N/158 L” for cHA-C, and “G/E/D/N/158 L and K/N163 P” for cHA-E (as illustrated in Figure 3). Further protein alignment using the Basic Local Alignment Search Tool (BLASTp) [35] of cHA-C and cHA-E, generated 85% and 82% similarities, respectively, to the HAs of A/swine/Tianjin/01/2004 (H1N1) and A/Taiwan/01/1986 (H1N1) viruses, confirming that both constructs are not of any random influenza virus subtype, but predominantly HA of the influenza H1 subtype.

Structures of cHA-C and cHA-E were predicted as haemagglutinins with close resemblance to the structure of a type 16 haemagglutinin, 4f23, on the protein database. Amongst other alterations, key amino acids within the receptor binding domain were slightly altered from typical amino acids on H1 HAs as indicated.

### 3.3. cHA B-Cell Epitope and Antigenic Predictions

**Figure 4:**
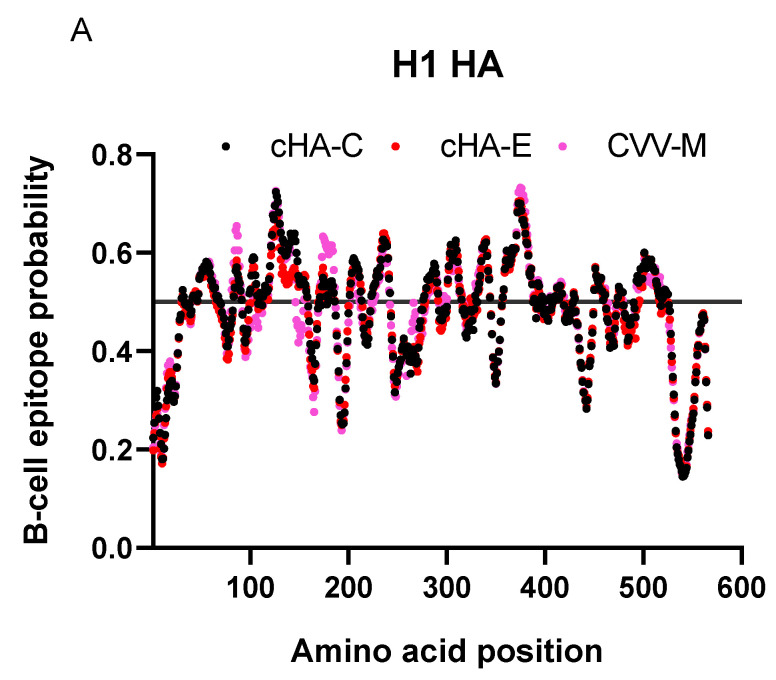

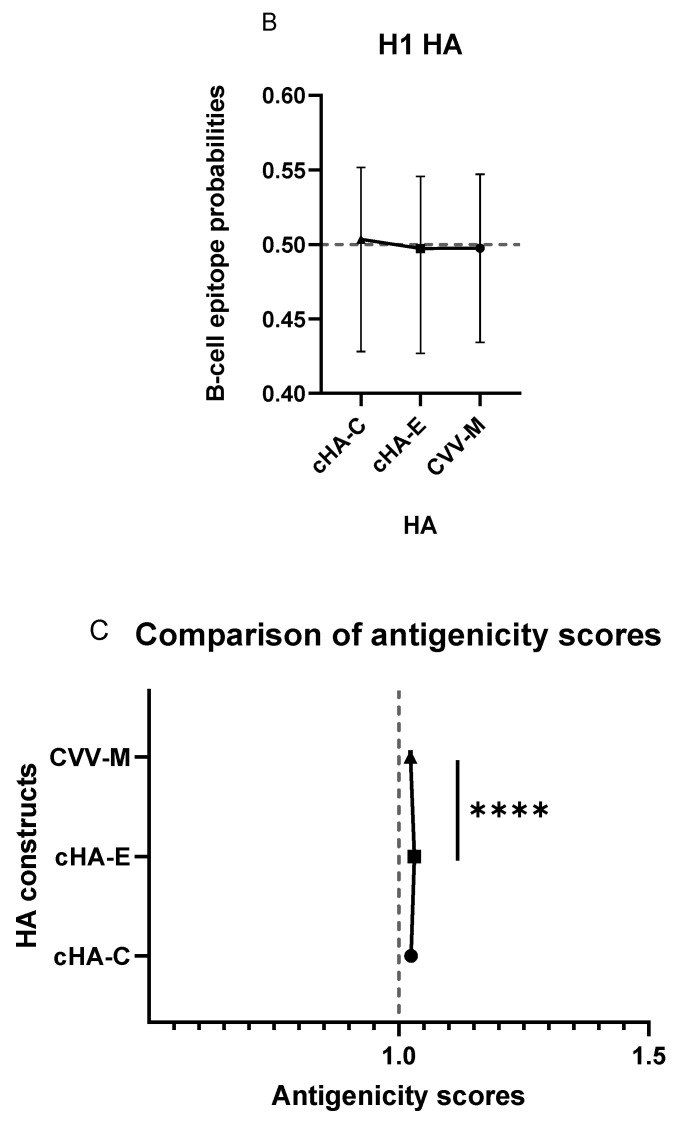
B-cell epitopes and antigenicity predictions on the H1 HAs. Here are charts describing B-cell epitope probabilities and antigenicity scores of the H1 HAs. (**A**) displays the distributions of the probable epitopes over the default threshold of 0.5; (**B**) represents the comparison of the medians of the B-cell epitope probability scores, using the Kruskal–Wallis test; (**C**) displays the antigenicity of the HAs given a default threshold of 1.000 antigenicity score: Means of the scores were compared using two-way ANOVA and *p*-values were corrected using Dunnett’s multiple comparisons test *p* < 0.0005: ****.

An important factor for the design was to enrich B-cell epitopes and immunogenicity of the cHAs by the introduction of foreign sequences; hence, blending the consensus H1 HA with conserved sequences of H5 or H9 HAs, in an attempt to introduce conserved foreign epitopes. B-cell epitopes predicted were slightly higher for the cHA-C and cHA-E versus the control HA, CVV-M (HA of the 2018/2019 Michigan vaccine strain—KY117023) (Figure 4A). Despite this observation, there were no significant differences amongst the median B-cell epitope probabilities of the cHAs and CVV-M (Figure 4B). Assessment of antigenicity of the cHAs based on Kolaskar and Tongaonkar algorithms at a threshold of 1.000 raised scores that were competitive between controls and the cHAs: both cHA-C and cHA-E recorded scores with means relatively higher than the CVV-M though all three crossed the set threshold; however, the mean of the antigenicity scores of cHA-E was significantly higher than those of both cHA-C and CVV-M (Figure 4C). Predicted antigenicity yielded four additional epitopes for cHA-C (three unique and one shared with cHA-E); cHA-E had six additional epitopes (five unique and one shared with cHA-C) and CVV-M had four additional epitopes (Table 2). At least the commonly shared epitope between cHA-C and cHA-E served as a key antigenic identifier of the cHAs. cHA-E was a relatively more antigenic HA construct, whereas cHA-C and CVV-M seem relatively similar due to possession of four antigenic epitopes each. cHAs designed, therefore, represent two extremes: low (cHA-C) and high (cHA-E) antigenicity, with CVV-M, categorizing with the lowly antigenic cHA-C.

### 3.4. Recombinant HAs Had No Detectable Hemagglutination Activity

**Figure 5:**
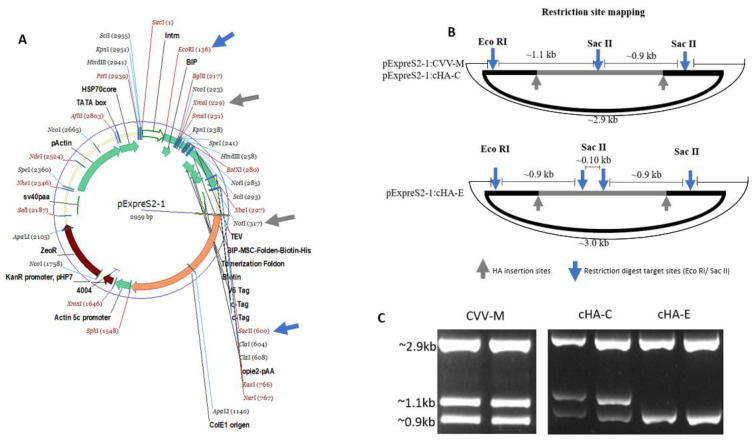
RFLP verification of the HA cloned onto the pExpreS2-1 plasmid. (**A**) Plasmid map detailing HA insertion sites between NotI and XmaI (grey arrows or grey outline B) and existing EcoRI and SacII restriction sites (blue arrows). (**B**) Representation of EcoRI and SacII restriction digest sites showing anticipated electrophoretic band patterns. (**C**) Restriction digest confirming expected electrophoretic band patterns: SacII cuts twice the plasmid with the CVV-M and cHA-C at similar sites and EcoRI cuts once, hence the three similar band patterns; cHA-E on the other hand, displays 3 SacII restriction sites, in addition to one EcoRI site, leaving two major bands. Primers were also designed to target the flanks of the HA insertion point on the plasmid. These primers as well as the cloned pExpreS2-1 plasmids were outsourced for sequencing by GeneArt Gene Synthesis (Thermo Fisher Scientific, UK). Returned sequences confirmed the HA orientation on the plasmid (not shown).

**Figure 6:**
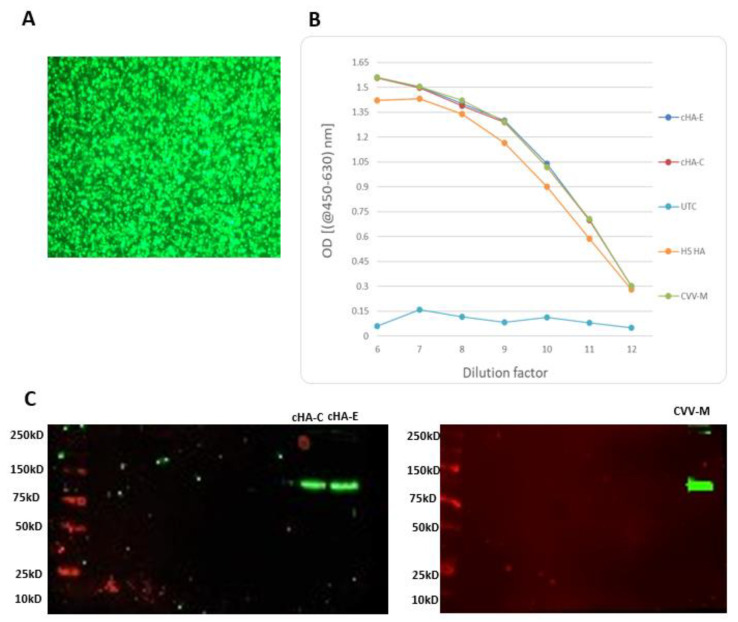
Confirmation of HA expression in S2 cells. Day three success of HA expression in supernatant of S2 cell culture confirmed by green fluorescent protein expression control (at a magnification of ×20) displaying about >90% transfection efficiency (**A**); ELISA, showing similar expression patterns for cHA-C, cHA-E and CVV-M controlled by an in-house H5 HA expressing-S2 cells, and supernatant from an un-transfected cell control (UTC) (**B**) or Western blotting showing blots of cHA-C, cHA-E and CVV-M at expected band sizes of approximately 75 kD (**C**).

The HA cloning processes were successful, and complete plasmid-HA construct was confirmed by restriction of digestion using EcoRI and SacII (Figure 5). The plasmids bearing HA constructs were used to successfully transfect S2 cells to a > 90% efficiency (Figure 6). Purified HA proteins (cHA-C, cHA-E, and CVV-M) were quantitated and adjusted to about 30 µg/mL, lacking haemagglutination capability: each of the expressed proteins recorded an *HA* titre < 2, whilst a stock of A/pheasant/New Jersey/1355/1998 (H5N2) laboratory isolate used as virus control had a titre of 128 *HA* units when mixed with 1% turkey red blood cells (Table 3).

### 3.5. Mice Were Not Previously Exposed to at Least the H1 Strains of Influenza Used

Mice were grouped (*n* = 5) into four groups: naïve control (U), cHA-C, cHA-E, and CVV-M. Before immunization, group-specific blood specimens pooled together were screened for exposure to two influenza A virus HA antigens by haemagglutination inhibition assay (HI). HI titres against the 2018/2019 WHO H1 HA antigen of A/Michigan/45/2015 (NYNC X-275 H1N1 pdm09) (X-275), serological kit antigen and the experimental challenge virus A/England/195/2009 (H1N1 pdm09) were <20, suggesting that these mice have not previously been infected with an influenza A virus (Table 4).

### 3.6. Stimulation of Mice with cHAs or CVV-M Induced Seroconversion

Sera from mice induced with constructs cHA-C, cHA-E, and CVV-M were assessed further for both seroconversion and cross reactivity against an H1 virus isolate (A/H1N1/England/195/2009) or H1 HA antigen (X-275). cHA-C, cHA-E, and CVV-M sera yielded HI titres of 320, 1280 and 640, respectively, against the H1 HA antigen; cHA-C, cHA-E, and CVV-M, each recorded a titre of 320 against the A/England/195/2009 (H1N1) pdm09. In all setups, sera collected from the naïve control (U) consistently recorded HI titres < 20, confirming that mice that received the HA constructs have been successfully immunized against the test influenza A viruses (Table 4).

#### 3.6.1. Anti-cHAs Antibodies Cross-React with Heterosubtypic H5N2 Virus

HI capacity of sera from mice vaccinated with the cHAs (cHA-C and cHA-E) in addition to the CVV-M control were assessed using H1N1 virus (X-275) and a heterosubtype virus isolate A/pheasant/New Jersey/1355/1998 (H5N2) (PNJ) acquired from the International Reagents and Resources (IRR). Except for the HA inhibition titre of sera from the naïve control group (U) that remained <20 HI units, all the other groups (CVV-M, cHA-C and cHA-E) generated a titre of 160 HI units each, suggesting that immunization with both the cHAs and CVV-M induced cross-reactive antibodies blocking the virus binding to the turkey red blood cells (Table 4). Furthermore, the observed cross-reactivity to an H5 virus by the cHAs, which was comparable to the control CVV-M, gives some credence to the design of the cHAs, in terms of comparability of antigenicity between these antigens and the control CVV-M. The cHAs by this display may possess broad cross-reactivity potential.

#### 3.6.2. Mice Vaccinated with cHAs Subunit Vaccines Showed Reduced Morbidity (Weight Loss Rebound) against the Lethal Dose H1N1 Pdm09 Virus Challenge

**Figure 7:**
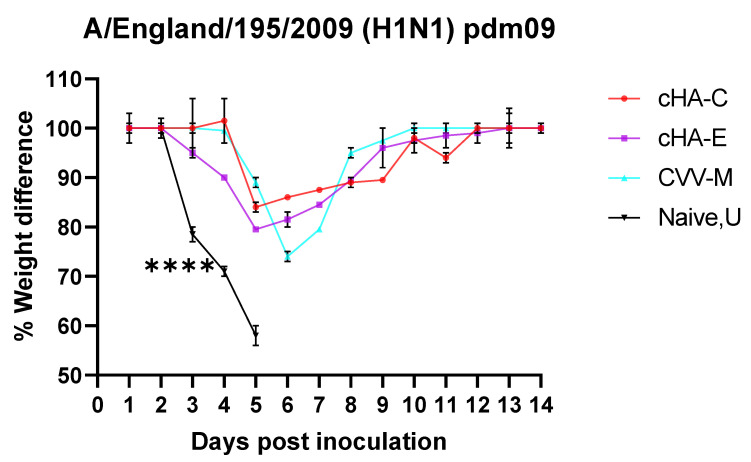
Weight monitoring of mice challenged with the A/England/195/2009. Summary of the weights of mice recorded over a period of 14 days during the influenza virus challenge; mice that were immunized with constructs cHA-C, cHA-E, and CVV-M before the H1N1 isolate (A/England/195/2009 pdm09) challenge, had their weights staying above 30%. The naïve group that received PBS before the challenge begun to lose weight steadily on day three, persisting until all mice euthanized by day five. Percentage weight differences were analysed by one-way ANOVA followed by Dunnett’s multiple comparisons test using the GraphPad prism 8.0.1. *p* < 0.00005: ****.

Weights of mice in vaccinated (cHA-C, cHA-E, CVV-M) and naïve control (U) groups were monitored for 14 days after challenged with A/England/195/2009 (H1N1) pdm09 virus. A relative drop in the weights of mice were observed across all groups. However, weights of mice immunized with cHA-C, cHA-E, CVV-M rebounded shortly following about 20% decrease in initial weights. Weights of mice in the naïve group, on the other hand, declined steadily below 30% by the 5th day, warranting euthanasia of all the naïve mice (Figure 7). Retrospective viral load assessments in one mouse per group by PCR during the relative drop in weight (around day three) was performed. This revealed higher virus titres (estimated by average threshold cycle, Ct values of approximately 26, 27, 24 and 18 for cHA-C, cHA-E, CVV-M, and U, respectively), giving a clear indication of relatively increased viral load amongst the naïve group, compared to the other HA-immunized groups. This is an indication that immunized groups presented variable resistance to the virus replication.

## 4. Discussions

Vaccination is one of the best means by which influenza infections can be controlled [36]. However, influenza viruses are always evolving, and this makes available vaccines lose their effectiveness. This is because there exists an arms race between influenza virus evolution and updating vaccines carrying optimal cross-reactivity against contemporary circulating strains [37,38]. Therefore, constantly updating vaccines with relatively suboptimal efficacy would not be ideal in the continuous fight against influenza epidemics or any unforeseen future pandemic. The way forward is to devise new strategies to circumvent scientific and technical barriers, such as the challenges in generating novel conserved antigenic epitopes that are potentially immune correlates of protection, and new immunological methods for screening these epitopes [39,40].

One opinion is that the active vaccination against influenza could partly be the problem for the induction of selective immune pressures driving the evolution of influenza viruses as a result of exposure to induced antibodies [38]. Antigenic imprinting is another challenge misleading immunity to current influenza infection or vaccination. Current vaccination attempts, thereby, seem superficial at increasing resistance to infection as immunity is short-lived and wanes away. However, the desire remains for the availability of a potential universal vaccine candidate that may offer a minimum of one-year-long protection with at least 75% efficacy in the control of symptomatic influenza caused by influenza A H1 and H3 virus subtypes [41]. Vaccines such as the computationally optimized broadly reactive antigen (COBRA) generation has shown potential, in that a single representative antigen that retains specific cross-reactive epitopes for H1, H3 and H5 [42,43,44] could protect against other strains. Chimeric HA-based vaccines have equally been useful in the robust induction of anti-stalk antibodies with broader cross-reactivity [45]. This study was a relatively simpler approach to explore the combinatorial effect of optimizing HA antigens through concurrent consensus sequence generation and chimeric HA formation.

Chimeric HA sequences (cHA-C and cHA-E) were designed predominantly to retain highly conserved amino acid sequences of the H1 HA, and this was confirmed using BLAST, which was confirmed with a minimum of 80% similarity to H1 HA sequences in the NCBI database. Of note, the structural change of antigenic epitope may affect the antiviral immune response against the target virus. The cHAs demonstrated structural similarity to a model influenza A HA subtype 16—a member of the group 1 HA to which H1, H5 and H9 HAs belong, whose crystal structure enabled visualization of the structures of the cHAs designed. Clearly key sequences of the H1 globular head domains, in addition to others spanning the complete structure, were slightly altered due to the introduction of foreign amino acids into key polymorphic regions. Immense editing could have thus accounted for the unsuccessful detection of haemagglutination activity. Unlike the constructs, cHA-C and cHA-E that were extensively altered, it is unclear why the candidate vaccine control HA (CVV-M) could also not haemagglutinate turkey red blood cells at 30 µg/mL. Perhaps, a relatively more concentrated version could have been successful. Nonetheless, since haemagglutination ability was not much of the focus of the work, no further assessment was performed.

Of interest are vaccine candidates with the potential to induce mostly humoral immune responses directed to all angles of the influenza A virus HA, especially those that target the relatively conserved regions on this glycoprotein. Such antibodies so triggered will target these highly conserved viral HA domains and could play crucial roles in facilitating protection against an infectious influenza virus via any of the antibody-dependent virus-arresting mechanisms such as antibody-dependent cellular cytotoxicity (ADCC), antibody-dependent cellular phagocytosis (ADCP) or direct virus neutralization as premised in a review by Kotey et al., 2019, amongst others [46]. Precise recognition and termination of an infectious virus will enable further fine-tune maturation of the immune system offering tremendous protection that may transcend any evolved strain.

Of note, immunization with viruses bearing mosaic HA has the potential to induce both HI-active (neutralizing) and stalk-reactive antibodies (due to recognition of both conserved globular HA head and stalk regions) vis-a-vis immunization with viruses bearing the chimeric HA that is associated with induction of mostly non-neutralizing stalk-directed antibodies (due to the varied globular HA head regions leading to a phenomenon termed as “stalk focusing”) [11,47]. cHAs in this study were also designed to be an intermediate between mosaic and chimeras, in that, all polymorphic regions on the complete length of the H1 HA have been substituted with amino acids from H5 (cHA-C) or H9 (cHA-E) HAs. It is thus expected that the cHAs generated will enhance the induction of both neutralizing (head directed) and non-neutralizing (stalk-directed) antibody responses. Seroconverted serum against CVV-M, cHA-C and cHA-E drawn from mice during the third week exhibited haemagglutination inhibition titres of 640, 320 and 1280, respectively, against H1 HA antigens of influenza A/Michigan/45/2015 NYNC X-275 (H1N1) pdm09 (X-275). These titres seem to correspond greatly with their predicted antigenicity scores—a parameter that was meant to estimate how much immune response would be triggered towards each of the constructs, and is observed, from highest to least, as cHA-E > CVV-M ≥ cHA-C. This is further supported by the observation of additional linear epitopes, six on cHA-E and four each present on cHA-C and CVV-M, but perhaps on the condition that the additional epitopes on CVV-M are relatively more immunogenic than those on cHA-C, hence a two-fold HI titre reduction of cHA-C (compared with CVV-M). Unsurprisingly, each of the expressed constructs exhibited a similar neutralization capacity (HI titres of 320 each) against the strain used subsequently for the challenge experiment, i.e., the A/England/195/2009 (H1N1) pdm09. Furthermore, for each of the constructs, some cross-reactive neutralization was observed against an H5N2 virus (A/pheasant/New Jersey/1355/1998 (PNJ)), with each HA construct (cHAs/CVV-M) producing an HI titre of 160, supporting the existence of some commonly conserved epitopes in all HAs belonging to the HA group 1. Although not many viruses were available for HI assessment employing the immune cHAs sera, HI activity against PNJ provides some data inferring a possibility of developing group-specific vaccines based on the cHAs. Cross-reactivity observed here, thus, demonstrates the need to scale up to assess more viruses with differing HA types in the future.

Mice that were immunized prior to the virus challenge regained weight quickly with no or minimal display of symptoms and remained stable throughout the 14 days of observation, unlike the naïve controls that were euthanized due to steady decline in weight past 30% by day 5. Protective antibodies triggered due to immunization were robust enough to restore stability in weight, confirming the protective capability of the cHAs. Though there were not any significant differences in the extent of weight drop and regain between the cHAs and the CVV-M, examination of the lungs of a mouse from each group during the drop in weights around day 3 revealed that viral load was much lower in the cHAs compared with the CVV-M (data not shown). Again, statistical differences amongst viral loads could not be inferred due to analysis in a single mouse per group, nevertheless, the trends were in favor of the cHAs, indicating protection of challenged mice as a result of a robust corresponding immune response. Further studies will be required to understand the nature of antibodies induced by these cHAs, as well as to investigate the presence of other non-neutralizing antibody protective mechanisms (whether by ADCC or ADCP or any others).

Although further work is required to assess cHAs-induced immunity to many other HA types, as well as examine the mechanisms of protection, work discussed here advances alternative means by which the conserved HA sequences (spanning the globular head and stalk) of the seasonal influenza A viruses could be harnessed to induce cross-reactive antibodies. This approach has the potential to be used for the development of a candidate universal influenza vaccine. Outcomes of this study further enhance our understanding how a cHA-based candidate vaccine had been improved for the development of a universal vaccine candidate on which clinical trials had documented desirable outcomes [12].

## 5. Conclusions

Identification of a universal influenza vaccine candidate has been rather perplexing. Recent isolations of broadly reactive antibodies, in addition to other diverse means of inducing these groups of antibodies have, however, given hope to identifying universal candidate antigens. This study further demonstrates that, perhaps, fine-tuning cHAs may improve the breadth of antibody responses to influenza A viruses bearing similar conserved sites.

## Figures and Tables

**Table 1:** Structural and functional predictions.

HAs	Predictions	
	Structural	Functional
	TM-Score	SIA-Binding C-Score
cHA-C	0.831	0.77
cHA-E	0.836	0.7
A/Michigan/45/2015 (H1N1) pdm09-like virus (CVV-M)	1	1

Note: Here is the figurative representation of the predictions made on the structures and functions of the cHAs to a typical HA molecule. Whilst TM scores provide inference into structural similarity of a query protein sequence to a hit template and ranges in likelihood from 0 to 1, the C-score, also ranging from 0 to 1 (or 0% to 100%), infers on functional similarity of a query protein sequence. CVV-M was assumed to be 1 for both the TM-score and C-score, whereas cHA-C and cHA-E were each about 80% structurally similar and had 70% potential to be similar in function as typical HAs.

**Table 2:** Unique and shared epitopes in the H1 HAs.

cHA-C				cHA-E				CVV-M			
Start	End	Peptide	Length	Start	End	Peptide	Length	Start	End	Peptide	Length
146	155	SSGVSAACSY	10	69	76	LGDCCTAG	8	93	98	WSYIVE	6
267	277	APEYAFALVRG	11	139	145	SWPVHYA	7	150	156	TAACPHA	7
347	352	FGAIAG	6	175	184	YPTLAASYAN	10	264	270	NLVVPRY	7
462	469	LYEKVKLQ *	8	247	253	YWTLLRP	7	289	295	VHDCNTT	7
				287	297	AVMCECEAKCQ	11				
				460	469	KKLYEKVKAQ *	10				

Note: Here are common or unique epitope-based peptides altered or generated on the cHAs compared to the control, CVV-M; peptides shared by the two cHAs, cHA-C and cHA-E are indicated with *.

**Table 3:** Turkey red blood cell haemagglutination assessment of cHAs and candidate vaccine virus HAs.

Expressed HA/Virus	HA Titre		
CVV-M	<2	<2	<2
cHA-C	<2	<2	<2
cHA-E	<2	<2	<2
Lab H5N2 (A/pheasant/New Jersey/1355/1998) virus isolate [PNJ]	128	128	128
PBS	<2	<2	<2

Note: Expressed cHAs and M (30 µg/mL) were challenged with 1.0% turkey red cells in the haemagglutination assay. Experiment was controlled with the expressed candidate vaccine HA (CVV-M), PBS and a laboratory H5N2 virus isolate (PNJ).

**Table 4:** Baseline/sero-converted blood assessment to influenza A (H1N1) pdm09.

Serum	Baseline				3D			
Virus	cHA-C	cHA-E	CVV-M	U	cHA-C	cHA-E	CVV-M	U
X-275	<20	<20	<20	<20	320	1280	640	<20
A/England/195/2009 (H1N1) pdm09	<20	<20	<20	<20	320	320	320	<20
PNJ	<20	<20	<20	<20	160	160	160	<20

Note: Here, seroconverted serum (3rd week serum drawn, 3D) is indicated to have generated significantly elevated haemagglutination inhibition titres compared with the baseline blood drawn. With the 3D, the H1-based cHAs (cHA-C and cHA-E) induced anti-cHAs antibodies that both reacted with H1-bearing viruses (the X-275 and A/England/195/2009) and an H5N2 virus (PNJ). A similar situation was observed for the control, CVV-M. The naïve control, U, remained constantly non-converted.

## Data Availability

All the available data have been reported in this manuscript.
